# The role of sirtuins in cellular homeostasis

**DOI:** 10.1007/s13105-016-0492-6

**Published:** 2016-05-06

**Authors:** Wioleta Kupis, Jan Pałyga, Ewa Tomal, Ewa Niewiadomska

**Affiliations:** 1 Department of Biochemistry and Genetics, Institute of Biology, Jan Kochanowski University, ul. Świętokrzyska 15, 25-406 Kielce, Poland; 2 Institute of Plant Physiology, Polish Academy of Sciences, Niezapominajek 21, 30-239 Kraków, Poland

**Keywords:** Deacetylation, Deacylation, Mono-ADP-ribosylation, Mammalian and plant sirtuins, Sirtuin activators and inhibitors

## Abstract

Sirtuins are evolutionarily conserved nicotinamide adenine dinucleotide (NAD^+^)-dependent lysine deacylases or ADP-ribosyltransferases. These cellular enzymes are metabolic sensors sensitive to NAD^+^ levels that maintain physiological homeostasis in the animal and plant cells.

## Introduction

Acetylation, catalyzed by acetyltransferases that transfer an acetyl residue from acetyl-CoA to the ε-amino group of specific lysine residues in histones and other proteins, is responsible for chromatin activation and regulation of metabolic pathways. A reverse process, the removal of acetyl group from the lysine of acetylated proteins, requires the participation of enzymes known as lysine deacetylases (KDACs). In general, the lysine deacetylases were divided into four classes: class I, II, III, and IV [49]. Since a yeast transcriptional repressor Sir2 (silent information regulator 2) is a founding member in the class III deacetylases, the homologue proteins in other organisms have been named sirtuins.

Sirtuins constitute a highly conserved family of deacetylases that depend on the oxidized form of nicotinamide adenine dinucleotide (NAD^+^) [6]. Seven homologs of yeast Sir2 (SIRT1–7) which share a conserved catalytic domain have been identified in mammals [23]. Sirtuins differ in subcellular localization, enzymatic activity, and targets (Table 1). SIRT1, SIRT6, and SIRT7 are nuclear proteins while SIRT2 is mainly a cytoplasmic protein but it can translocate into nucleus as well [59]. SIRT3, SIRT4, and SIRT5 are mitochondrial sirtuins [50].

**Table 1:** Subcellular location, enzymatic activity, function, and selected non-histone target substrates for mammalian sirtuins

Sirtuin	Subcellular localization	Enzymatic activity	Function	Target substrates	References
SIRT1	Nucleus Cytoplasm	Deacetylase	Formation of facultative and constitutive chromatin Mitochondrial biogenesis Fatty acid oxidation Regulation of cholesterol and bile acid homeostasis	p53, FOXO1/3, NF-κB, CRTC2, PGAM-1, PGC1α, SREBP, LXR, FXR, LKB1	[8, 39]
SIRT2	Cytoplasm Nucleus (transiently)	Deacetylase Demyristoylase	Cell cycle regulation Promotion of lipolysis in adipocytes Tumor suppression/promotion Neurodegeneration	α-Tubulin, FOXO1, FOXO3, p300	[17, 57]
SIRT3	Mitochondria	Deacetylase Decrotonylase	Regulation of mitochondrial activity Protection against oxidative stress Tumor suppression	LCAD, ACS2, SOD2, IDH2, HMGCS, OTC, SOD2, subunits of the electron transport chain and ATP synthase	[3, 8, 50, 53, 66]
SIRT4	Mitochondria	ADP-ribosylase Deacetylase Lipoamidase	Glucose metabolism Amino acid catabolism Tumor suppression	IDE, ANT2, ANT3, GDH, MCD, PDH	[44, 50, 66]
SIRT5	Mitochondria Cytoplasm Nucleus	Deacetylase Demalonylase Desuccinylase Deglutarylase	Urea cycle Fatty acid metabolism Amino acid metabolism	CPS1, UOX	[18, 28, 46, 50]
SIRT6	Nucleus	ADP-ribosylase Deacetylase Deacylase	Genomic stability/DNA repair Glucose and lipid metabolism Inflammation	HIF1α, PARP1, TNFα, GCN5	[32, 37, 40]
SIRT7	Nucleus (nucleolus)	Deacetylase	Ribosome biogenesis Tumor promotion	RNA polymerase 1	[4, 58]

*ACS2* acyl-CoA synthetase 2, *ANT* adenine translocator, *CPS1* carbamoyl-phosphate synthase 1, *CRTC2* CREB-regulated transcription coactivator 2, *FXR* farnesoid X receptor, *GCN5* general control non-repressed protein 5 (an acetyltransferase), *GDH* glutamate dehydrogenase, *HIF-1* hypoxia-induced factor 1, *HMGCS* 3-hydroxy3-methylglutaryl CoA synthase 2, *IDE* insulin-degrading enzyme, *IDH2* isocitrate dehydrogenase 2, *LCAD* long-chain-specific acyl coenzyme A dehydrogenase, *LKB1* liver kinase B1, *LXR* oxysterol receptor, *MCD* malonyl CoA decarboxylase, *OTC* ornithine transcarbamoylase, *PDH* pyruvate dehydrogenase, *PGAM-1* phosphoglycerate mutase-1, *PGC-1α* peroxisome proliferator-activated receptor (PPAR) γ coactivator 1α, *SOD2 (MnSOD)* mitochondrial manganese superoxide dismutase, *SREBP* sterol regulatory element-binding protein

Sirtuins play an important role in the regulation of cellular homeostasis, in particular metabolism [30], inflammation [27], oxidative stress [55], and senescence [53]. It is believed that activation of sirtuins may be advantageous not only in metabolic diseases such as type 2 diabetes and obesity, but also in neurodegenerative diseases [17]. This is in part because the sirtuins stimulate the activity of mitochondria, the energy centers of the cells, and mitochondrial proteins, preventing physiological changes underlying many pathological conditions [30].

## Structure of sirtuins

**Fig. 1:**
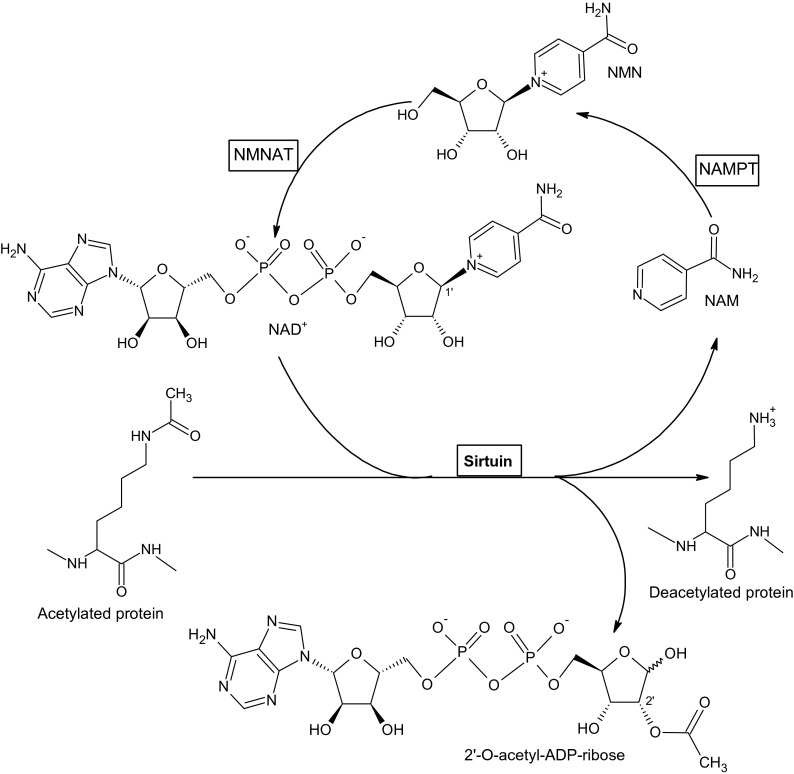
Deacetylation of acetylated proteins by sirtuins and the conversion of resulting nicotinamide into NAD^+^. *NAM* nicotinamide, *NMN* nicotinamide mononucleotide. The enzymes involved are *boxed*: *NAMPT* nicotinamide phosphoribosyltransferase, *NMNAT* nicotinamide mononucleotide adenylyltransferase

All sirtuins possess a conserved catalytic NAD^+^-binding domain, consisting of about 275 amino acids, which is flanked by the N- and C-terminal sequences of variable length [54]. The N- and C-terminal extensions are the targets for posttranslational modifications that can affect the functions of sirtuins [22]. A larger sirtuin domain consists of α/β Rossmann-fold structure that is a characteristic for NAD^+^-binding proteins while a smaller domain includes a zinc-binding module containing three-stranded antiparallel β-sheet and a variable α-helical region [21]. Cofactor (NAD^+^)-binding loop region, connecting the small domain to the Rossmann-fold structure, consists of four loops forming an extended cleft that acts as the enzyme active site. Both NAD^+^ and acetylated lysine-containing substrates bind to this pocket [54]. The NAD^+^-binding site can be divided into three regions: site A, binding site for adenine-ribose moiety; site B, nicotinamide-ribose binding region; and site C, nicotinamide moiety binding site [54]. In the presence of acetylated lysine, NAD^+^ can undergo a conformational change bringing the nicotinamide group in the proximity to the C site where it can be cleaved. After nicotinamide cleavage, the acetyl carbonyl oxygen of the acetyl-lysine nucleophilically attacks the carbon C1′ of the ribose to form a first intermediate between the two substrates which is the 1′-O-alkylamidate. Then, the intermediate is hydrolyzed to produce a deacetylated polypeptide and 2′-O-acetyl-ADP-ribose [54, 56] (Fig. 1).

## Enzymatic reactions of sirtuins

Nicotinamide adenine dinucleotide is an essential cofactor for electron transfer in an intermediate metabolism that is converted into a reduced form NADH [6]. The sirtuins can act as the sensors of cell metabolic state because they are sensitive to the intracellular ratio of NAD^+^/NAM [6] and the changes in NAD^+^ levels will directly affect sirtuin activity and substrate preference [20]. One may envision that the sirtuins may transmit the signal of changes in the metabolism to chromatin through deacetylation of histones and other chromosomal proteins [59], ultimately leading to alterations in gene expression.

**Fig. 2:**
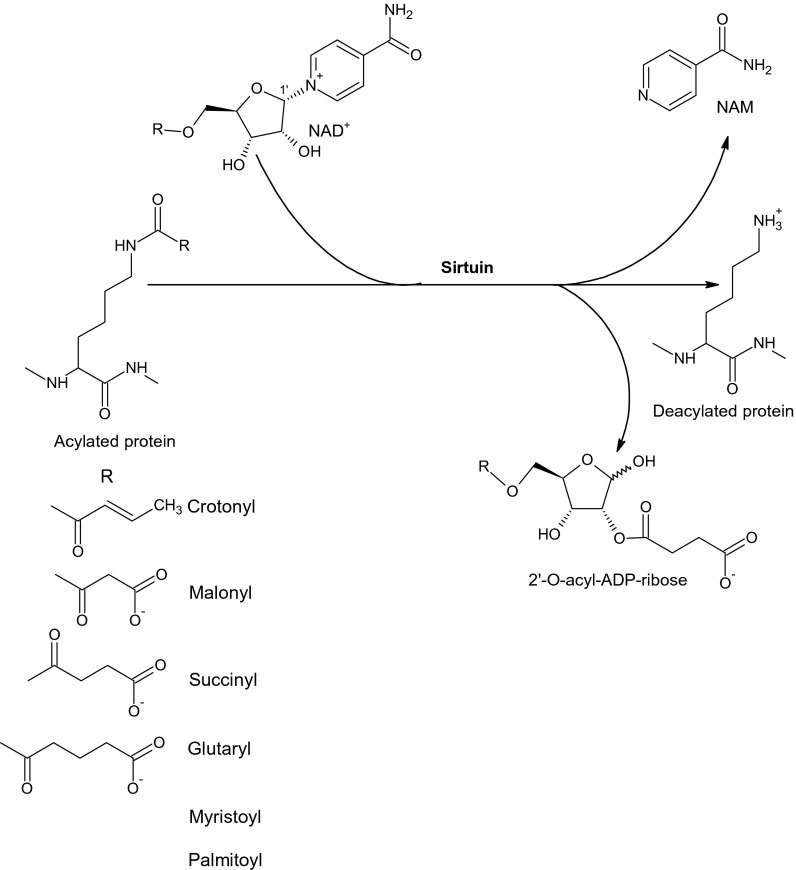
Deacylation reaction performed by sirtuins; 2′-O-succinyl-ADP-ribose is shown as the product of deacylation reaction catalyzed by SIRT5. The long-chain fatty acid moieties can be removed by SIRT2 or SIRT6

**Fig. 3:**
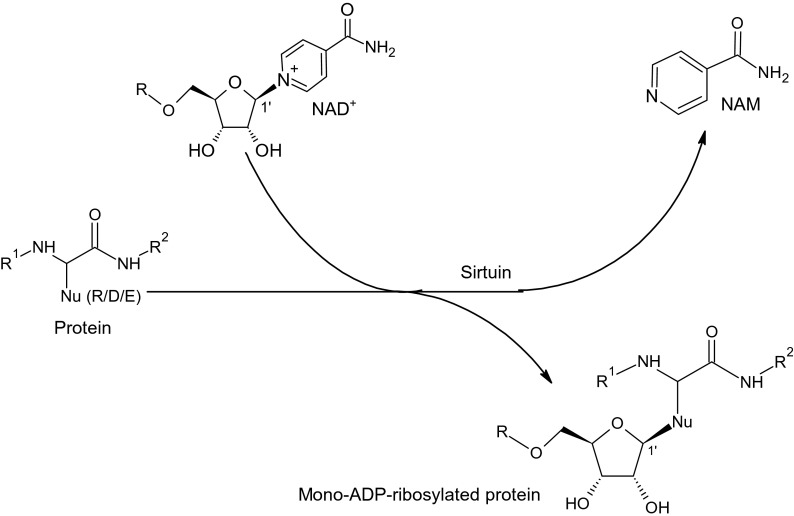
ADP-ribosylation conducted by SIRT4 and SIRT6. *Nu* nucleophilic group of the amino acid side chain, *R/D/E* Arg/Asp/Glu; the letter *R* in the NAD^+^ and ADP-ribosylated protein denotes ADP moiety

In addition to the deacetylation of nucleosomal histones and metabolic enzymes, the sirtuins may also exhibit other activities. Although SIRT1 and SIRT2 could decrotonylate histone peptides in vitro [19], SIRT3 is the major in vivo decrotonylase, specifically involved in the regulation of H3K4cr [3, 51]. SIRT2 exhibits activity for the removal of long-chain fatty acyl groups [41] with a higher catalytic efficiency for a myristoyl group than that for the acetyl group [57]. It turned out that SIRT4 does not show histone deacetylase activity and acts primarily as a mitochondrial ADP-ribosyltransferase [26]. SIRT4 is also a cellular lipoamidase that regulates the pyruvate dehydrogenase complex activity [44]. SIRT5 may act as a demalonylase, desuccinylase, and deglutarylase [18, 28] leading to the removal of acid acyl moieties linked to the lysine residues in the protein (Fig. 2). SIRT6, which exhibits deacetylase and fatty deacylase activities [19, 32], also functions as a nuclear mono-ADP-ribosyltransferase [40]. The latter reaction involves the transfer of a single ADP-ribose moiety from NAD^+^ to an acceptor amino acid residue (arginine, asparagine, aspartate, glutamate) in various proteins to form N- or O-glycosidic bonds, depending on a nucleophilic group in the amino acid side chain [10] (Fig. 3). In general, sirtuins can act as ADP-ribosyltransferases or protein deacylases that use either unmodified proteins as a substrate (ADP-ribosylation by SIRT4 and SIRT6) or proteins modified with acetyl, malonyl, succinyl, and glutaryl [28, 30] or other acyl residues such as crotonyl [3, 51] and fatty acid residues [32, 41].

## Sirtuin subcellular localization and function

This section only briefly discusses the broad functional diversity of sirtuins, and much more information on the topic can be found in the following reviews [5, 6, 8, 12, 15, 17, 27, 30, 37, 39, 50, 55]. Mammalian sirtuins occupy different cellular locations, act on several substrates, and may perform various functions (Table 1). SIRT1 is the best characterized protein of the sirtuin family. It participates in the formation of both constitutive and facultative chromatin and appears to play a dual role as a suppressor or promoter during carcinogenesis [5]. It regulates a number of pathways associated with normal metabolism and functioning of individual organs in mammals [30, 39]. In liver, SIRT1 promotes gluconeogenesis by deacetylation and activation of PGC1α (peroxisome proliferator-activated receptor γ (PPARγ) coactivator 1α) and FOXO1 (forkhead box A1) and inhibits glycolysis by deacetylating and suppression of the glycolytic enzyme phosphoglycerate mutase 1 (PGAM-1) [8]. Similarly, in the skeletal muscle, SIRT1 deacetylates PGC1α and interacts with AMP-activated protein kinase (AMPK) to form a reciprocal positive regulating loop in which AMPK activates SIRT1 by increasing the level of NAD^+^ due to the upregulation of the gene encoding an NAD^+^ synthetic enzyme nicotinamide phosphoribosyltransferase (NAMPT) while SIRT1 activates AMPK by deacetylation of its activator, liver kinase B1 (LBK1).

SIRT2 which is present predominantly in the cytoplasm colocalizes with microtubules and deacetylates the main component of microtubules, α-tubulin, at lysine 40 [15]. During cell cycle progression from G_2_ to M, SIRT2 translocates to the nucleus to deacetylate histone H4 at lysine 16 leading to chromatin condensation during metaphase. SIRT2 also deacetylates the transcription factors FOXO1 and FOXO3 and lysine residues in the catalytic domain of histone acetyltransferase p300 [17].

SIRT3 positively regulates the activity of mitochondria by deacetylation and activation of several components of the electron transport chain complexes I and II and acetyl-CoA synthetase (ACS) [53]. SIRT3 also affects a defense against oxidative stress protecting cells from reactive oxygen species (ROS). Indeed, during calorie restriction, SIRT3 activates superoxide dismutase 2 (SOD2), a key mitochondrial antioxidant enzyme [11].

SIRT4, located in the mitochondrial matrix, is ubiquitously expressed in kidney, heart, brain, liver, and pancreatic β cells. SIRT4 suppresses the secretion of insulin in response to glucose and interacts with insulin-degrading enzyme (IDE) [50]. By transferring the ADP-ribose residue, the enzyme inactivates glutamate dehydrogenase (GDH) that converts glutamate to α-ketoglutarate in the mitochondria, thus leading to reduced ATP synthesis [66].

SIRT5 is localized in the mitochondrial matrix, mainly in brain, heart, liver, and kidney. SIRT5 deacetylates carbamoyl synthetase 1 (CPS1) which catalyzes the first rate-limiting step of the urea cycle. The CPS1 deacetylation by SIRT5 increases the activity of the enzyme. It has been shown [18, 50] that CPS1 is deacetylated during calorie restriction, and its activity increases on low-calorie diet. An increase in urate oxidase (UOX) deacetylation and activity was detected in mice overexpressing SIRT5 in the liver [46, 50].

Poly-(ADP-ribose) polymerase 1 (PARP1) that stimulates the repair of DNA damage in response to oxidative stress is ADP-ribosylated by SIRT6 to promote its poly-ADP-ribosylation activity [37]. SIRT6 is capable of removing fatty acyl residues from the lysines 19 and 20 of tumor necrosis factor α (TNFα) to regulate its release [32]. Overexpression of SIRT6 in male mice significantly extended their life, and these individuals, as compared to the wild type ones, had the elevated levels of insulin-like growth factor 1 (IGF1) [34].

SIRT7 participates in the transcriptional activation catalyzed by RNA polymerase I and III [35, 58] and may interact with hypoxia-induced factors HIF-1α and HIF-2α to lower their expression [31]. The enzyme has been shown [4] to maintain malignant transformation of the cells through H3K18 deacetylation. SIRT7 is also a dynamic nuclear regulator of mitochondrial homeostasis acting on GABPβ1 (GA binding protein β1), a master regulator of mitochondrial biogenesis and function [52].

## Modulators of sirtuin activity

Investigations conducted in mice have shown [50] that activation or inhibition of sirtuins can alleviate pathological conditions. Therefore, measures have been taken to identify compounds that can inhibit or activate specific sirtuins. Most of these studies concern modulators of SIRT1, the main nuclear sirtuin. However, since the importance of the other sirtuins continues to grow, therefore, they may prove to be equally attractive targets for the modulators [43].

### Sirtuin activators

Polyphenols are plant secondary metabolites and represent a large group of compounds of variable structural complexity with aromatic rings containing one or more hydroxyl groups. A growing number of reports suggests [2] that polyphenols from food (for example, resveratrol, quercetin, and catechins) are capable of changing epigenetic state of the cell. These compounds alter among others KDAC activity thus leading to the activation or silencing of specific genes [2].

It has been shown [16] that resveratrol (3,5,4′-trihydroxystilbene), found *inter alia* in red wine and grape skins, increases the affinity of SIRT1 for acetylated peptide substrates. This sirtuin activator binds to the enzyme-substrate complex and lower *K*_m_ for the acetylated substrate without affecting the *K*_m_ for NAD^+^ or *V*_max_ [16]. Resveratrol promoted deacetylation of PGC-1α by SIRT1, leading to a reduction in body weight and insulin resistance, and an increase in motor function and survival in mice with high fat diet-induced obesity.

In addition to resveratrol, several other small molecules (SRT1460, SRT1720, SRT2183) that activate SIRT1 were described [16]. These compounds were found to be approximately 1000-fold more potent than resveratrol. Among them, SRT1720 appeared to be the most promising SIRT1 activator, the administration of which improved glucose homeostasis, increased sensitivity to insulin, and improved mitochondrial function in type 2 diabetes mouse models [16]. The neuroprotective properties of SRT2104, an activator of SIRT1, were reported in mouse models of Huntington disease [33]. Finally, NAD^+^-dependent sirtuin activity has also been shown to increase when cells or animals are treated with NAD^+^ precursors such as niacin, nicotinamide, nicotinamide riboside or nicotinamide mononucleotide [6].

### Sirtuin inhibitors

It has been shown [47] that sirtinol inhibits the activity of sirtuins and reduces inflammation in capillary endothelial cells of the skin and is therefore a likely target in the treatment of skin disorders. Cambinol is an example of a competitive inhibitor that competes with acetylated polypeptides, suggesting that it binds close to the substrate binding site (as does splitomycin, another β-naphthol-containing sirtuin inhibitor). The fact that β-naphthol compounds bind to other site than NAD^+^ causes that they are less toxic [43]. Suramin, a urea derivative, shows similar characteristics and competes for binding with both NAD^+^ and the acetylated lysine of the substrate [62]. However, it has a neurotoxic activity which greatly limits its therapeutic use [7].

Indole derivative EX-527, a selective SIRT1 inhibitor, easily penetrates into cells. Administration of this compound strongly increased the acetylation of p53 protein at K382 following the induction of DNA damage in human mammary epithelial cells and some tumor cell lines [7]. In contrast to the β-naphthol derivatives, it binds to the NAD^+^ binding site of sirtuins. Other example of inhibitory indole derivative is oxyindole (selective for SIRT2 in vitro) that inhibits α-tubulin deacetylation in MCF-7 mammary cells [43]. A SIRT2 inhibitor AK7 exhibits neuroprotective effects in models of Parkinson disease [9].

Thiourea-based compounds called tenovins can also attenuate the activity of sirtuins. Tenovins are highly hydrophobic, and this property hampers or even precludes their use in vivo. However, a promising prospect involves synthesis of tenovin-6, a more water-soluble analogue [45], which effectively restricts the development of tumor in a mouse model of melanoma [43]. A new class of SIRT1 inhibitors with the scaffold of benzofuran-3-yl-methanone has been identified [61]. The inhibitors bind to the C-pocket of SIRT1, forming hydrophobic interactions with the enzyme. Since C-pocket is the site where the nicotinamide moiety of NAD^+^ binds and the hydrolysis takes place, binding the inhibitor to the C-pocket would block the transformation of NAD^+^ to productive conformation and thus inhibit the deacetylase activity. An analogue with hydroxyls at *ortho* and *meta* positions, (2,5-dihydroxyphenyl)(5-hydroxy-1-benzofuran-3-yl)methanone, which is a non-competitive inhibitor for acetylated peptides and a mixed competitive inhibitor for NAD^+^, is a more potent SIRT1 inhibitor than nicotinamide [61]. A different compound, inauhzin, inhibits the activity of SIRT1 and efficiently reactivates p53 to promote a p53-dependent apoptosis of human cancer cells without causing visible genotoxic stress [63].

## Plant sirtuins

Despite many reports on sirtuins in many species including fungi and mammals [24], the function of these enzymes in plants is still poorly understood. In general, plant histone deacetylases were classified on the basis of their homology to the yeast HDACs into three families: (1) RPD3/HDA1, (2) SIR2, and (3) HD2 [48]. In comparison with the fungi (five genes) and animals (seven genes), the number of *SIR2* genes in plants is strongly reduced and, in most cases, two genes were detected [42]. In *Arabidopsis*, 18 KDACs, including two sirtuins—SRT1 and SRT2 (Table 2)—have been revealed [29]. Initial studies indicated inhibitory role for sirtinol (sirtuin inhibitor) in the development of the hypocotyl and root vascular tissue in *Arabidopsis* seedlings [25] thereby suggesting that SRT1 and SRT2 might have a role in auxin signaling [25, 29]. Subsequent work has shown [14] that *SIR* genes were a part of the pathway for sirtinol metabolism to the active auxin, 2-hydroxy-1-naphthoic acid. While in mammalian mitochondria three types of sirtuins were identified with different physiological functions [38, 50], the *Arabidopsis* genome encodes only one mitochondrial sirtuin, SRT2, with seven splice variants identified [36]. Western blot analysis showed the presence of two mature SRT2 proteins—a shorter SRT2A with a mass of 31 kDa and longer SRT2B (35 kDa), which differ mainly in the C-terminal domain sequence. SRT2 protein is located in the inner mitochondrial membrane and acts on specific proteins associated with the membrane (including ATP synthase and ATP/ADP transporters) [36].

**Table 2:** Members of the sirtuin family in plants

Species	Sirtuin	Function	References
*Arabidopsis thaliana*	AtSRT1 AtSRT2	Plant tissue development Stress response regulation Mitochondrial energy metabolism	[25, 29, 36, 60]
*Oryza sativa*	OsSRT1 OsSRT2	Transposon silencing Metabolism and stress response regulation	[65]
*Solanum lycopersicum*	SlSRT1 SlSRT2	Regulation of gene expression	[64]
*Vitis vinifera*	VvSRT1 VvSRT2	Leaf senescence Stabilization of chromatin structure and regulation of gene expression	[1, 13]

The members of SIR2 deacetylase family have been also identified in other plant species, for example, in rice *Oryza sativa* [65], tomato *Solanum lycopersicum* [64], and grapevine *Vitis vinifera* [1, 13] (Table 2).

The rice genome comprises at least 19 genes encoding KDACs which, as in the case of *Arabidopsis thaliana*, belong to three families [65]. OsSRT1 (also called SRT701), homologous to mammalian SIRT6, is expressed in the nucleus, while OsSRT2, a homologue of mammalian SIRT4, is located in mitochondria. The OsSRT1 is involved in the deacetylation of histone H3 at lysine 9 that is present mainly at the 5′ ends of the genes, suggesting that the initiation sites for gene transcription can be targeted by the enzyme. The action of OsSRT1 has also been linked to transposon silencing [65].

Two proteins in the SIR2 family (SlSRT1 and SlSRT2) were identified in tomato flowers and cotyledons [64]. While the *SlSRT1* was expressed in the cell nucleus, the product of the gene *SlSRT2*, which is homologous to the mitochondrial *AtSRT2*, was identified both in the nucleus and cytoplasm. A distinct intracellular localization may suggest that different members of the tomato SRT family may play different roles [64].

*VvSRT1* and *VvSRT2* are members of the SIR2 family in the grape vine genome (*V. vinifera*). The *VvSRT1* transcripts in roots, leaves, flowers, and fruit and *VvSRT2* transcripts in leaves, flowers, and fruits were revealed [1]. It has been suggested [13] that VvSRT2 may be indirectly linked to chloroplasts activity and the regulation of chloroplast gene expression. Moreover, VvSRT2 reached its highest expression levels in the senescent red leaves of the grapevine, whereas the expression of VvSRT1 was not altered [13].

## Conclusions

Over the last several years, a lot of efforts have been made to better understand the mechanisms of sirtuin actions. Disturbances in energy metabolism, genome stability, response to cellular stresses, and lifespan shortening in mice lacking specific sirtuins demonstrate that these enzymes could contribute to the maintenance of cellular homeostasis in mammals. A modification of the sirtuin activity by small molecule activators or suppressors may provide new opportunities for the treatment of type II diabetes, obesity, and neurodegenerative diseases associated with aging or to clarify the role of sirtuins in the carcinogenesis. To achieve these goals, a progress should be made in understanding the cellular effects of sirtuins as well as in identifying additional targets and modulators for these enzymes. Similarly, the exact function of plant SIR2 lysine deacetylases is not fully understood and further research is needed to explain their role.
